# Intracellular nanoparticles mass quantification by near-edge absorption soft X-ray nanotomography

**DOI:** 10.1038/srep22354

**Published:** 2016-03-10

**Authors:** Jose Javier Conesa, Joaquín Otón, Michele Chiappi, Jose María Carazo, Eva Pereiro, Francisco Javier Chichón, José L. Carrascosa

**Affiliations:** 1 Centro Nacional de Biotecnología (CNB-CSIC), Cantoblanco, 28049 Madrid, Spain; 2 ALBA Synchrotron Light Source, MISTRAL Beamline - Experiments Division, 08290 Cerdanyola del Vallès, Barcelona, Spain; 3 Unidad Asociada CNB-Instituto Madrileño de Estudios Avanzados en Nanociencia (IMDEA Nanociencia), Cantoblanco, 28049 Madrid, Spain

## Abstract

We used soft X-ray three-dimensional imaging to quantify the mass of superparamagnetic iron oxide nanoparticles (SPION) within whole cells, by exploiting the iron oxide differential absorption contrast. Near-edge absorption soft X-ray nanotomography (NEASXT) combines whole-cell 3D structure determination at 50 nm resolution, with 3D elemental mapping and high throughput. We detected three-dimensional distribution of SPIONs within cells with 0.3 g/cm^3^ sensitivity, sufficient for detecting the density corresponding to a single nanoparticle.

The recently increased use of superparamagnetic iron oxide nanoparticles (SPION) in nano-biomedicine for diagnosis, drug delivery and hyperthermia treatment^1^ has underlined the need for quantitative data of the interaction of these particles with cells at nanometric resolution beyond classical microscopy approaches and bulk assays. Several methods are used to correlate cell morphology with specific metal identification and quantitation, each optimized for a specific sample size window and resolution^2^. Light microscopy combined with cytological staining is rapid^3^, but resolution is very limited and bulk metal identification is possible only in few cases. Of the 3D X-ray approaches that can be used to quantify iron oxide nanoparticle distribution within a whole cell, 3D X-ray fluorescence (XRF) nanotomography^4^ provides the greatest chemical sensitivity but lacks cell structural detail. Alternatively, near edge X-ray absorption fine structure (NEXAFS)^5^ allows to differentiate between elemental species, but in the case of biological samples its three-dimensional counterpart prevented by radiation damage. Another alternative, which provides chemical and structural information at the same time, is 2D ptychography combined with 2D XRF that offers a way to study trace elements in their structural context^6^. 3D ptychography has recently been applied for imaging *Chlamydomonas* cells although with a resolution of 180 nm^7^. Nevertheless, this method requires collection times of hours^7^. An alternative well suited for biological samples, demanding much shorter exposure times, is near-edge absorption soft X-ray nanotomography (NEASXT), which can generate datasets in a few minutes with a sensitivity of 0.3 g/cm^3^, sufficient for detecting the density of a single nanoparticle, and with nanometric spatial resolution of 50 nm in 3D, while also providing a 3D ultrastructural cellular context^8^.

## Results

**Figure 1:**
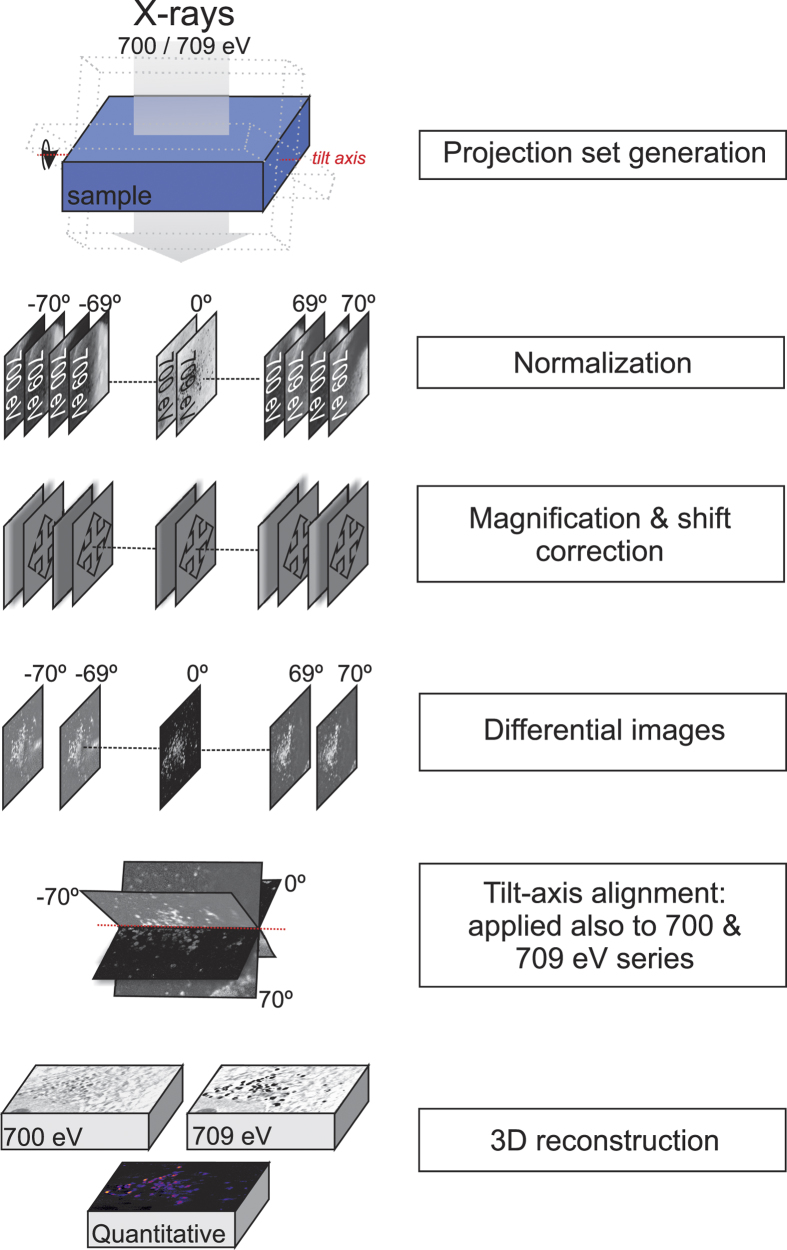
Near-edge absorption soft X-ray nanotomography (NEASXT) workflow.

We present an efficient, high-throughput method, NEASXT, which allows studying the variability of biological processes. In addition to adaptation of previous concepts, the workflow (Fig. 1) demands new image processing approaches to provide precise mass quantification by three-dimensional tomographic reconstructions in the whole cell.

**Figure 2:**
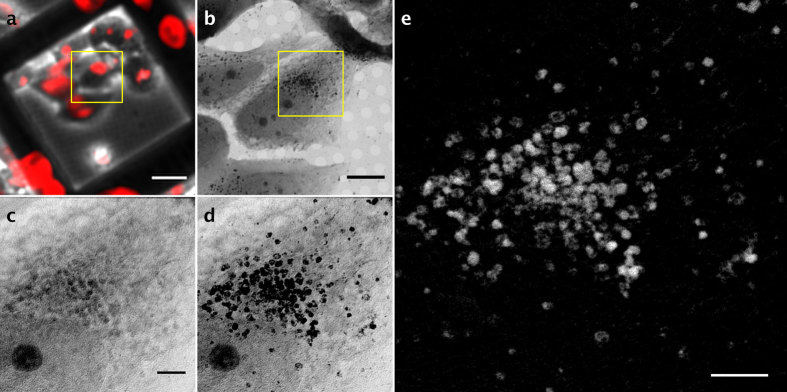
Correlative microscopy and differential tilt-series generation. (**a**) *In vivo* fluorescent image. Acidic organelles are labeled with Lysotracker (red). Scale bar, 25 μm. (**b**) Soft X-ray mosaic image (709 eV) from the area enclosed in the yellow square in (a). Scale bar, 8 μm. (**c,d**) 0° projection images of the area enclosed in the yellow square in (b) at 700 (c) and 709 eV (d). (**e**) Differential 0° projection image containing the SPION signal. Bar in **c–e**), 2 μm.

Human MCF-7 adenocarcinoma cells internalize superparamagnetic iron oxide (SPION) composed of mag-hemite^9^ *via* the endosomal pathway, which leads to endosome accumulation near the trans-Golgi network in the perinuclear area^10^ (MC, JJC, EP, MJR, KH, GS, FJC and JLC, in revision). MCF-7 were cultured on gold finder grids and incubated with SPION (cubes of 14 ± 1 nm side)^9^ functionalized with dimercaptosuccinic acid (0.2 mg/ml final concentration) for 24 h. Cells were labeled with Lysotracker for correlative optical fluorescence microscopy to track acidic organelles in which SPION accumulate (Fig. 2a), and then submitted to critical point drying to avoid water absorption beyond 535 eV. Grids were transferred to the Mistral beamline^11^ at the ALBA synchrotron to acquire tomographic series at −170 °C. The area of interest was first selected with the optical transmission microscope online inside the transmission X-ray microscope vacuum chamber. Correlation of *in vivo* optical fluorescence maps with X-ray projection mosaics of large areas (140 μm × 140 μm) allowed us to locate acidic vesicles inside cells (Fig. 2a). Tomographic acquisition was then set up to generate the datasets following the workflow shown in Fig. 1. Tomographic tilt-series were obtained using the 40 nm zone plate lens with a depth of focus (DOF) of 5.4 μm at 700 and 709 eV from −70° to 70° every 2° with 1 s exposure time, to yield 14.44 nm effective pixel size.

Using the iron L3 absorption edge, we acquired tilt-series at 700 eV (Fig. 2c) and 709 eV (Fig. 2d) to specifically detect the absorption changes corresponding to SPION, as the absorption corresponding to cellular components remained constant at these two energies. To facilitate two-dimensional projection alignment for generation of a differential tilt-series (i.e., subtraction of projection absorption images at each energy) and further 3D reconstruction, we acquired an image at each tilt angle for each energy (Fig. 1). Based on comparison of zero degree images before and after series acquisition, we observed no apparent radiation damage (Supplementary Fig. 1). We estimated the accumulated total X-ray dose to 2.5 × 10^8 ^Gy (considering a mean density of 1.35 g/cm^3^), well below the damage limit at our achievable resolution^12^.

**Figure 3:**
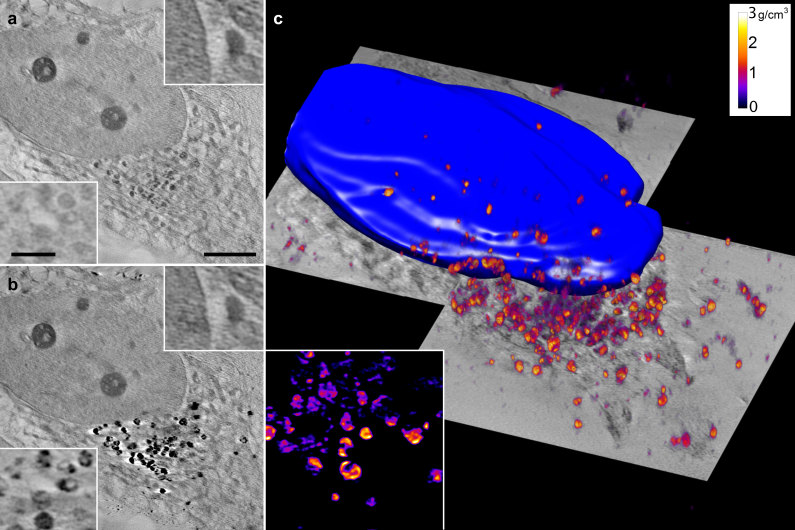
NEASXT slices from reconstructions at 700 and 709 eV and volumetric representation of iron oxide densities. (**a,b**) Cellular context resolved by reconstructing single energy tilt-series. Insets show the same regions at different energy. (**c**) Volumetric representation of iron oxide densities within the cells, forming clusters near the nucleus. The inset shows internal substructures of the clusters in a slice of the volume. Scale bar, 2 μm. Inset scale bars, 0.5 μm.

As the absorption changes at these energies are due to SPION absorption within the cell, the difference between the two aligned stacks allowed us to extract the specific SPION signal at each projection (Fig. 2e). This differential tilt-series was deconvolved (JO, EP, COSS, RM and JMC, in preparation), aligned to the common tilt axis and reconstructed using an ART (algebraic reconstruction technique) algorithm to recover the absorption coefficients (Fig. 3). We calculate a resolution of 50 nm using noise-compensated leave-one-out^13^ algorithm (NLOO) as well as following the Rayleigh criteria (Supplementary Fig. 2). To reconstruct the cell structural context, we used the two individual tomograms at 700 and 709 eV with the same tilt-alignment transformations. Voxel densities of the reconstructed differential volume were calculated, resulting in a 3D density map of the SPION in the cell.

Both volumes imaged at 700 and 709 eV showed cell ultrastructure that included the nucleus, heterochromatin, cytoplasmic organelles such as mitochondrion or vesicles, filaments, and the cell membrane (Fig. 3a,b; Supplementary Fig. 3). SPION differential volume showed clusters of different densities distributed near the nucleus (Fig. 3c and Supplementary movie) and in the same area as the optical fluorescence signal from the acidic organelles (Fig. 2a). Within the reconstructed volume, SPION clusters showed internal substructures (Fig. 3) with a mean iron oxide density of 0.9 g/cm^3^ (0.3–2.8 g/cm^3^) and a maximum diameter of 1.2 μm (Fig. 3c and Supplementary movie). This method allowed us to estimate the total SPION mass within a cell volume of ~90 μm^3^ as 11.5 pg, which accounted for a missing wedge correction factor.

## Discussion

NEASXT combines 3D ultrastructure determination at the whole cell level with specific elemental mapping, using differential absorption contrast within radiation damage limit. The implementation of this method allows for high throughput, an aspect that is essential to deal with the high variability of biological samples.

The method presented in this work is able to extract valuable biological information in the cellular context in contrast to bulk techniques commonly used for iron quantitation (inductively coupled plasma mass spectrometry, ICP-MS)^3^. Other 3D X-ray techniques which can provide quantification within the cellular context at 50 nm resolution, such as XRF, NEXAFS or ptychography, are hampered by 1) long acquisition times preventing statistical analysis, 2) the dose given to the sample, or 3) the need of chemical and structural information at the same time.

This study of SPION-treated cancer cells using NEASXT shows the possibility of precise identification and 3D mapping of iron oxide nanoparticles within cells at 50 nm resolution (see Supplementary Fig. 2) or better (depending on the zone plate lens used). Still, the resolution of the reconstructed volume is affected by the angle step chosen between projections (i.e. 2 degrees to reduce radiation damage), misalignments of the tilt series as well as the missing wedge. Another factor that limits the resolution is the out of focus information. For instance, the sample thickness (3.5 μm) was smaller than the DOF (5.4 μm), but the large field of view (14 μm * 14 μm) implies that part of the sample is out of focus at high tilt angles. All these effects are evident when comparing the 3D achieved resolution of 50 nm with the 2D measured one, 28 nm (JO, EP, COSS, RM and JMC, in preparation).

We have calculated a sensitivity of 0.3 g/cm^3^ thanks to the signal recovery achieved by projection deconvolution included in our data processing workflow (Supplementary Fig. 4) (JO, EP, COSS, RM and JMC, in preparation). This sensitivity is sufficient for detecting the differential absorption of a single nanoparticle even if we are not able to resolve it spatially since the SPION size is smaller than the spatial resolution.

The NEASXT approach could be valuable for *in silico* or *in vitro* evaluation of distinct SPION types to select those better suited for hyperthermia treatment, drug delivery or imaging diagnostics. The study of nanoparticles generated with other metal oxides with absorption edges within the water window would allow efficient, quantitative metal maps in near-native frozen whole cells, a major step towards implementing intracellular tags for correlated light, electron and X-ray microscopies.

## Methods

### Cell growth and sample preparation

MCF-7 cells were cultured on gold electron microscopy finder grids (G200F1, Gilder) coated with a thin layer of Quantifoil (R2/2, Quantifoil Micro Tools) in Dulbecco’s modified Eagle’s medium (DMEM) supplemented with 10% fetal calf serum. Cells were maintained in an atmosphere of humidified air with 5% CO_2_ at 37 °C. Depending on cell type, gold grids occasionally required additional functionalization with poly-Lys, poly-ornithine or another adherent biocompatible molecule. SPION (cubes of 14 ± 1 nm side)^9^ are surrounded by a layer of dimercaptosuccinic acid.

SPIONs (final concentration 0.2 mg/ml) were incubated for 24 h. Samples were stained with 100 nM Lysotracker Red DND-99 and 14.3 μM DAPI (both from Molecular Probes, Life Technologies) in DMEM. Samples were imaged by visible light fluorescence microscopy in a Leica DMI 6000B (Leica Microsystems) using a digital camera (Hamamatsu) with a 20x objective lens. To avoid cell overlap at high tilt angles during tomogram acquisition, grids with ~70% cell confluence were selected. Cells were fixed in 2.5% glutaraldehyde, 4% paraformaldehyde in phosphate-buffered saline (PBS) for 5 min, washed twice in PBS and dehydrated in an ethanol series (30, 50, 70, 80, 90 and 100%, 10 min each), then transferred without exposure to air to the critical point drier sample chamber (CPD030, Bal-Tec).

### X-ray microscopy

Samples were transferred to the Mistral beamline at the ALBA synchrotron^11^ in a dried atmosphere (N_2_), and inserted into the microscope operating at −170 °C to reduce radiation damage. Tilt-series were recorded using a zone plate objective with an outermost zone width of 40 nm (DOF 5.4 μm at 700 eV and 709 eV) to obtain a larger field of view (14.2 μm × 14.1 μm.). Tomographic series were collected from −70° to 70° with a 2° step and 1 s exposure time to reduce the dose.

To achieve precise alignment, it was necessary at each tilt angle to acquire an image at 700 eV followed by another at 709 eV. Projection images were recorded with an effective pixel size of 14.44 nm. The monochromatic photon flux impinging on the sample was on the order of 4.6 × 10^10^ photons/s. To obtain the complete cell volume (Fig. 3), two datasets of two tomograms at 700 and 709 eV were acquired, as dictated by cell size.

### Data processing

Image stacks were normalized with flat field images using a program implemented in XMIPP3.1^14^; beam current, and exposure time were corrected as well as the modulation transfer function (MTF), measured experimentally following the procedure described by Otón *et al.*^15^. Images were then deconvolved using a Wiener filter (Supplementary Fig. 4) (JO, EP, COSS, RM and JMC, in preparation). This deconvolution process is not part of current SXT data processing workflows, but was essential for correct mass quantification. Alignment of tilt pairs (images at 700 and 709 eV at each angle) were corrected for magnification, followed by X and Y shift alignment using mutual information scoring function implemented in EFTEM-TomoJ software^16^. To speed up acquisition time and eliminate possible hysteresis, the CCD-sample distance was fixed while changing photon energy. This involved an off-line software magnification correction prior to reconstruction. Acquired transmission projection images at both energies were converted to absorption images by applying the logarithm and difference between them. The results are projection images with an iron oxide-specific signal, which were aligned by IMOD^17^ using intrinsic sample features as fiducial markers. The same alignment transformations were applied to 700 and 709 eV datasets. We used 15 iterations of the ART algorithm with a relaxation factor of 0.01 implemented in TomoJ^16^ to recover absorption coefficients from the projection images while maintaining proportionality in all reconstructed voxels. For background equalization, differential iron-oxide tilt-series was submitted to an additional iteration using the BgART algorithm^16^ with a relaxation factor of 0.01 and a k value of 4.4. Resulting tomograms had a voxel size of 14.44 nm × 14.44 nm × 14.44 nm.

Tomogram resolution was calculated by NLOO^13^ and by Rayleigh criteria (Supplementary Fig. 2). Volume visualization and segmentation were carried out in Amira (FEI), Chimera^18^ and ImageJ^19^.

To extract densities, and hence maghemite mass per voxel from the iron oxide signal within the reconstructed volumes, we applied the Beer-Lambert Law as follows:


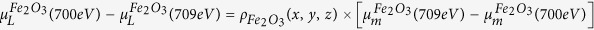


where μ_L_ is the linear absorption coefficient, μ_m_ the mass absorption coefficient and ρ the material density. The values in the resulting 3D density maps were corrected by 0.88 for a tilt-series from −70° to 70°, to approximately compensate for the missing wedge effect. The compensation factor was estimated by reconstructing phantom volumes with different missing wedges and comparing the absorption coefficients obtained with those in the phantom reconstruction without a missing wedge. To establish the NEASXT sensitivity, we calculated the signal to noise ratio of the reconstructed volume prior to BgART algorithm step. We equalized the background at a density in which the signal to noise ratio was equal to one, then, we defined the sensitivity as the minimum density that we can detect with a probability of 99.9% in the final reconstructed volume.

## Additional Information

**How to cite this article**: Conesa, J. J. *et al.* Intracellular nanoparticles mass quantification by near-edge absorption soft X-ray nanotomography. *Sci. Rep.* **6**, 22354; doi: 10.1038/srep22354 (2016).

## Supplementary Material

Supplementary Information

Supplementary Movie 1
