# Supplementary material for: Intracellular nanoparticles mass quantification by near-edge absorption soft X-ray nanotomography
Source: Sci Rep. 2016 Mar 10;6:22354. doi: 10.1038/srep22354 (PMC4785355; doi:10.1038/srep22354)
Supplement: Supplementary Information [file srep22354-s1.pdf]

Supplementary Material

Intracellular nanoparticles mass quantification by near-edge absorption soft X-ray nanotomography

Jose Javier Conesa[1], Joaquín Otón[1], Michele Chiappi[1], Jose María Carazo[1], Eva Pereiro[2], Francisco Javier Chichón[1]* and José L. Carrascosa[1,3]*

[1]Centro Nacional de Biotecnología (CNB-CSIC), Cantoblanco, 28049 Madrid, Spain

[2]ALBA Synchrotron Light Source, MISTRAL Beamline - Experiments Division, 08290 Cerdanyola del Vallès, Barcelona, Spain

[3]Unidad Asociada CNB-Instituto Madrileño de Estudios Avanzados en Nanociencia (IMDEA Nanociencia), Cantoblanco, 28049 Madrid, Spain

Supplementary Figure Legends

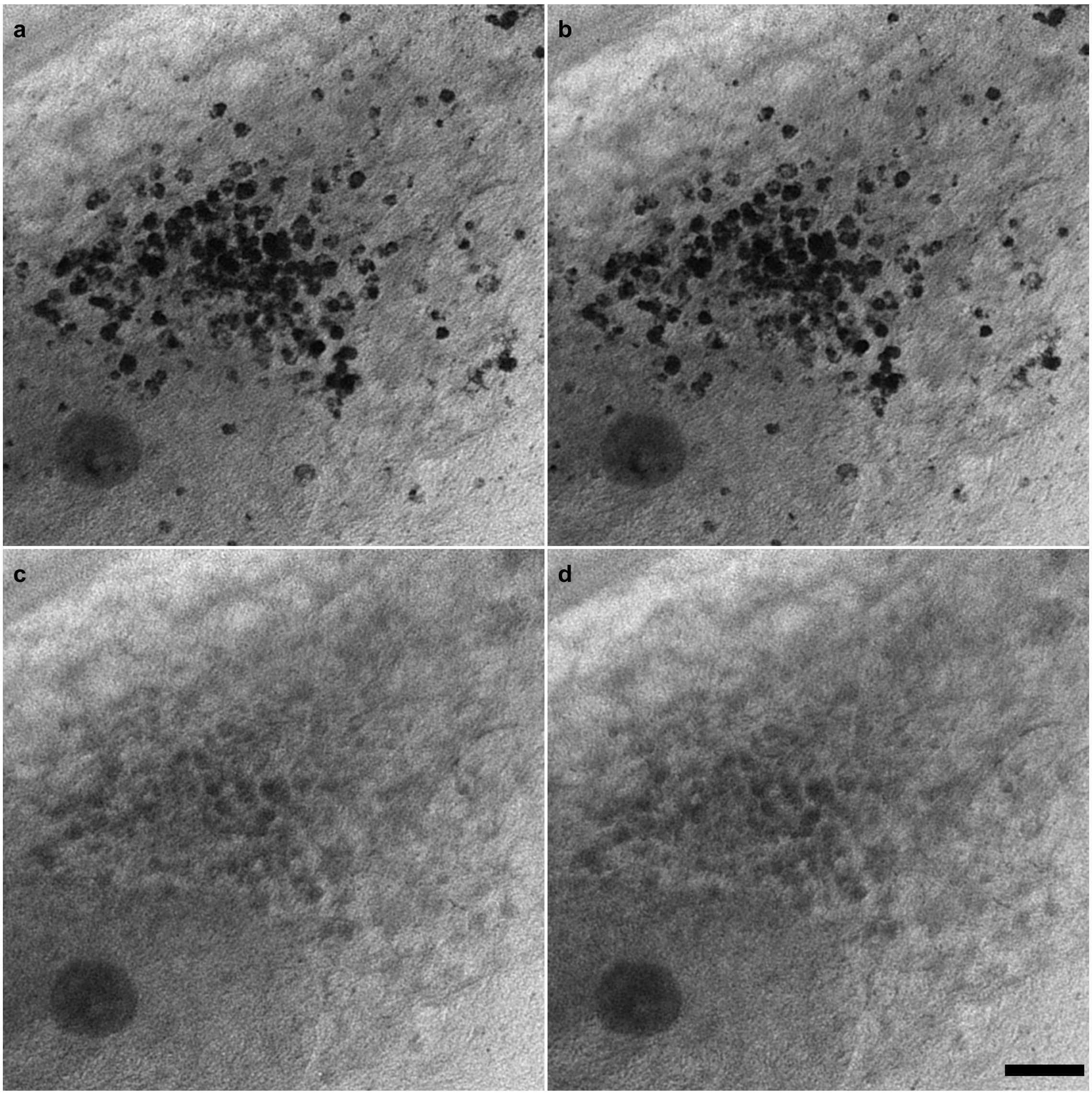

Supplementary figure 1. Comparison of transmission images before and after tilt series acquisition. Image a) and c), acquired at 709 and 700 eV photon energy respectively, before tilt-series acquisition compared with image b) and d), acquired at 709 eV and 700 eV photon energy respectively, after. No apparent radiation damage is observed by comparison of before and after tilt-series acquisition images. Scale bar, 2 μm.

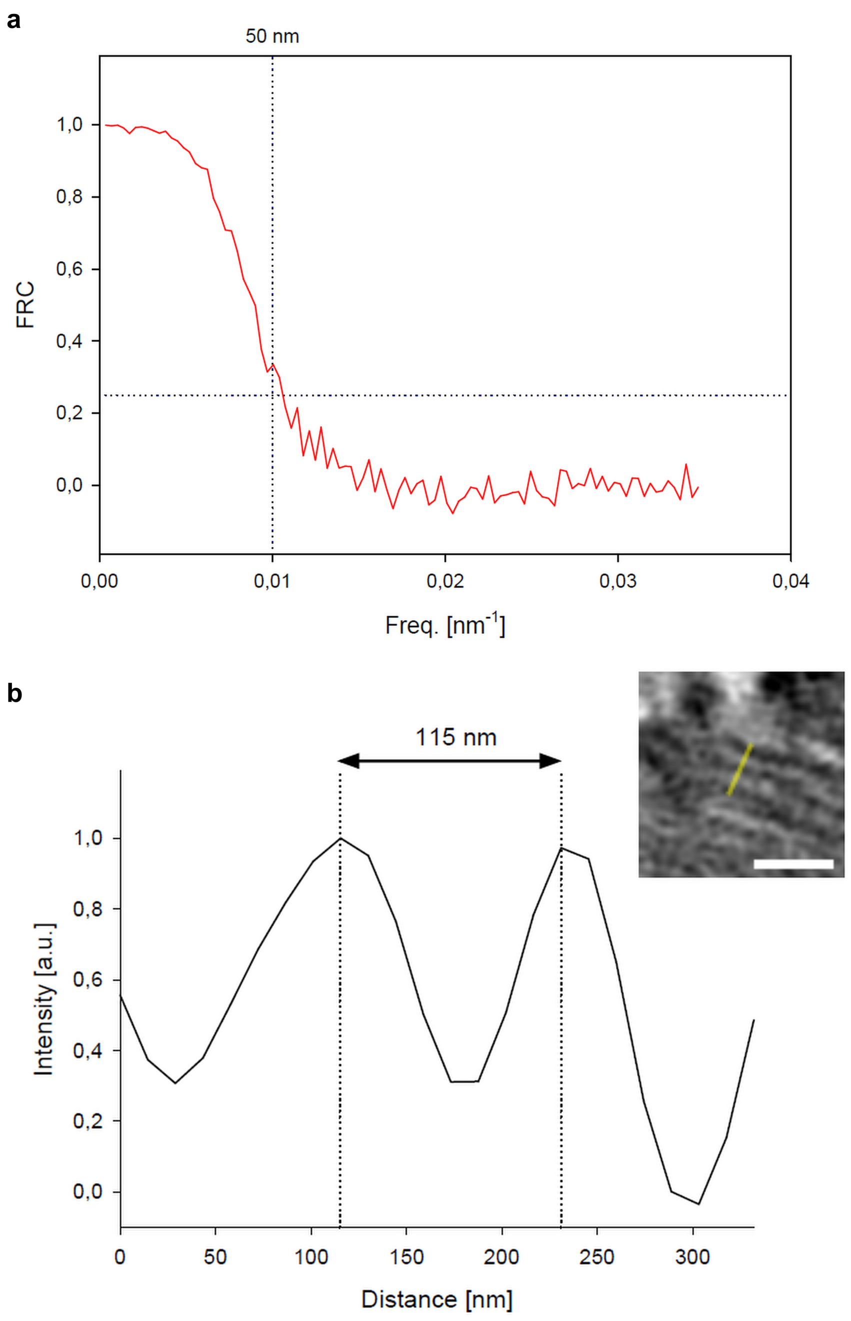

Supplementary figure 2. NEASXT resolution evaluation. a) Resolution curve calculated using NLOO algorithm, which corresponds to 47 nm 3D resolution. b) Resolution estimation following Rayleigh criterion. The resolution corresponds to 57.5 nm half pitch. Inset in upper right corner shows the area where the linear plot was calculated. Curve was calculated averaging five central tomogram series images resolution curves to decrease noise. Inset bar, 500 nm.

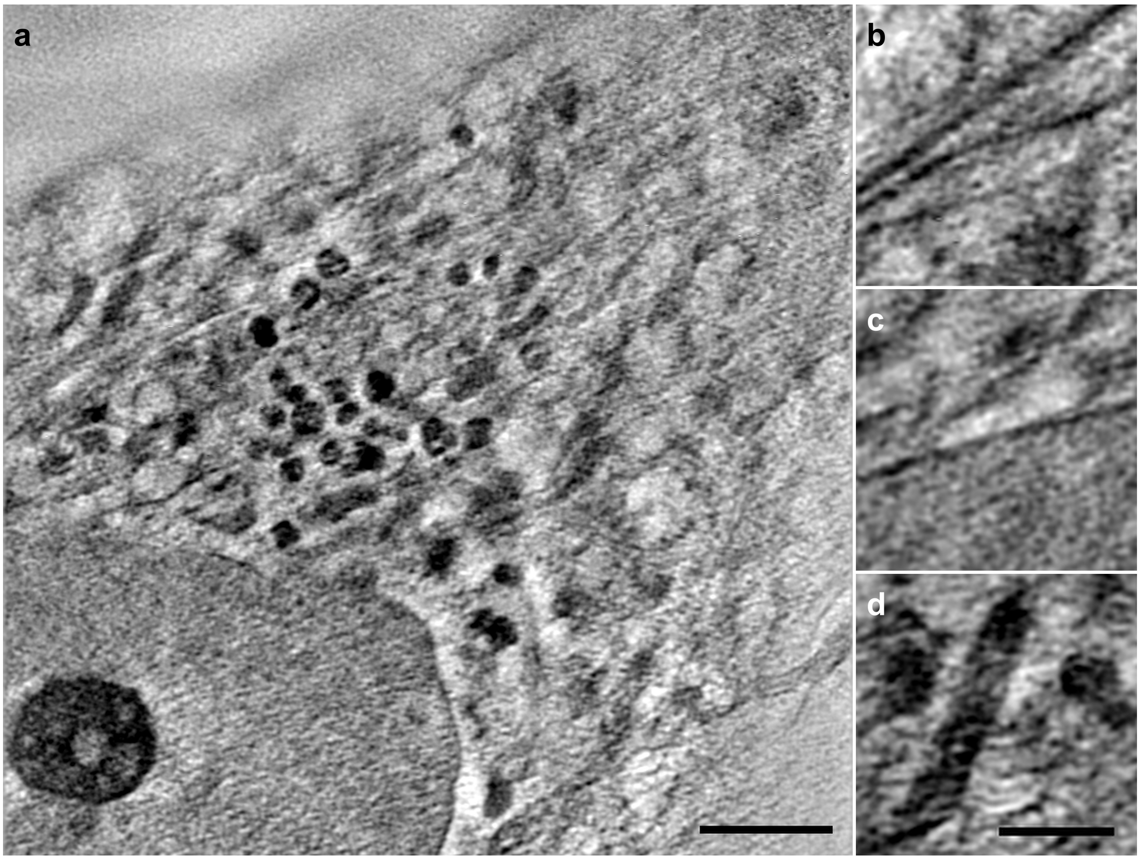

Supplementary figure 3. Tomogram sections of MCF-7 cells incubated with SPION. a), central tomogram section of a tomogram where cellular nucleus (N), heterochromatin (H) and vesicles (V) are distinguished. b), c) and d) show filaments, nuclear membrane and mitochondrion respectively. Scale bar in a, 2 μm. Scale bar in b, c and d, 1 μm.

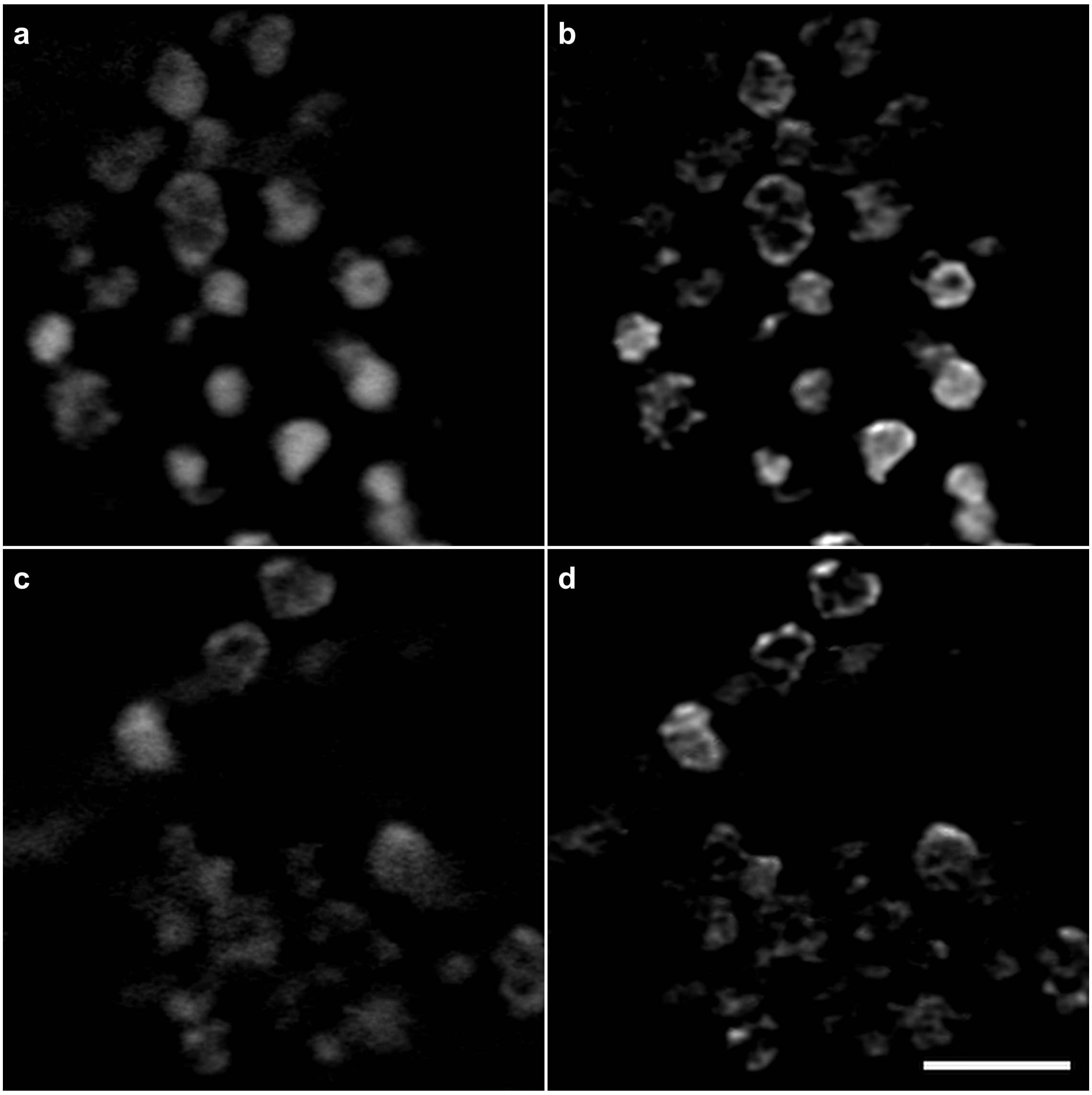

Supplementary figure 4. Comparison between non-deconvolved and deconvolved iron oxide signal tilt-series reconstructions. Left panels show non-deconvolved tomograms sections. Left panels show deconvolved tomograms sections. a-b) and c-d) correspond to same area. Scale bar, 1 μm.

Supplementary movie. NEASXT slices from reconstructions at 700 and iron oxide densities. Cellular context at 700 eV. Color-coded iron oxide densities within the cells, forming clusters near the nucleus.
